# OA15 A case of cardiac manifestation of systemic sclerosis

**DOI:** 10.1093/rap/rkac066.015

**Published:** 2022-09-28

**Authors:** Cara Pattiata, Luke Sammut

**Affiliations:** Portsmouth Hospitals NHS Trust, Portsmouth, United Kingdom; Portsmouth Hospitals NHS Trust, Portsmouth, United Kingdom

## Abstract

**Introduction/Background:**

A 45-year-old lady, originally from Peru, with a background of diffuse cutaneous systemic sclerosis presented with shortness of breath on exertion, frequent palpitations, non-exertional chest tightness, and fatigue.

**Description/Method:**

In 2011, she presented with inflammatory arthritis as well as Raynaud’s phenomenon. Her anti-nuclear antibody screen showed a positive anti-SCL-70 and anti-U3RNP. Over the years she developed pulmonary hypertension, renal involvement (proteinuria), dysphagia, cutaneous involvement (sclerodactyly, digital ulcers, telangiectasia) and interstitial lung disease. She was commenced on methotrexate and prednisolone. Her pulmonary hypertension was controlled on macitentan and sildenafil. Her pulse was 112 beats per minute, regular with a blood pressure of 168/85 mmHg. She had a loud P2 and a parasternal heave. She had sparse basal crepitations. She had mild pitting oedema to her knees. Her JVP was not raised. Her weight was 38kg.

Her right heart catheterisation in April 2021 reported a mean pulmonary artery pressure of 24mmHg and pulmonary vascular resistance of 255 ARU. A computerized tomography (CT) scan of her chest showed known interstitial lung disease and no pulmonary embolism. Her lung function tests were stable. A cardiovascular magnetic resonance imaging (CMR) guided cardiac catheter in April 2021 showed scleroderma involvement of the heart, with mild pericardial effusion and elevated native myocardial T1 and T2. Left ventricular ejection fraction was normal. The impression was that of scleroderma with myocardia involvement. Her immunosuppression was escalated to cyclophosphamide. A cardiac MRI in September 2021 showed improvements in T1 and T2 mapping parameters. She completed 4 cycles of cyclophosphamide (15mg/kg). However, due to gastrointestinal side effects (nausea and vomiting) and a diagnosis of latent tuberculosis in February 2022, cyclophosphamide was stopped. In March 2022, her repeat CMR showed that the T1 and T2 mapping had slightly increased and presented again with chest pain and palpitations. Following a multi-disciplinary team discussion, the patient was treated with rituximab 500mg two weeks apart with 100mg of methylprednisolone as premedication prior to the infusions.

**Discussion/Results:**

Although subclinical cardiac involvement in scleroderma is common, only 10 to 30% of patients are symptomatic. Our patients complained of non-exertional chest pain. Macrovascular coronary disease had been excluded; the cause of her chest pain was likely secondary to microvascular dysfunction. In patients with cardiac scleroderma, the mechanism of coronary disease is secondary to myocardial inflammation and repeated microvascular ischaemia. This results in reperfusion defects secondary to necrosis, in turn driving myocardial fibrosis. In the late stages, fibrosis results in left ventricular diastolic dysfunction, a complication related to increased mortality.

Supraventricular tachycardia is one of the manifestations in conduction abnormalities in cardiac scleroderma: others include conduction defects and bradyarrhythmias. As with our patient, tachyarrhythmias can be associated with symptoms such as generalised fatigue, shortness of breath, and exercise intolerance.

Another complication found on imaging was a small pericardial effusion. This is the most common pericardial disease in scleroderma. For most, pericardial effusions are asymptomatic; however, it can be associated with complications such as tamponade.

Whilst cyclophosphamide is a common treatment choice for scleroderma with cardiac involvement, evidence for this is limited to case series due to its rarity. Additionally, it carries significant toxicity: Our case developed significant nausea and vomiting following her third and fourth cyclophosphamide infusions and this was discontinued also in light of her diagnosis of latent TB. Rituximab, the agent to which the patient was subsequently switched, similarly has a growing body of evidence in cardiac scleroderma. Since her rituximab infusions, our case has remained stable and she has noticed an improvement of her clinical symptoms.

**Key learning points/Conclusion:**

Cardiac scleroderma is associated with significant reduction in quality of life, morbidity, and mortality. It is often difficult to treat due to sparsity of high-quality evidence (as in other rare conditions) as well as the multiple organ systems and co-morbidities involved. This case illustrates the clinical manifestation of cardiac scleroderma and the difficulty in selecting appropriate immunosuppression medication.

